# The effectiveness of different teaching methods on medical or nursing students: Protocol for a systematic review and network meta-analysis

**DOI:** 10.1097/MD.0000000000021668

**Published:** 2020-10-02

**Authors:** Bei Yun, Qian Su, Yi-Tong Cai, Lian Chen, Chao-Ran Qu, Lin Han

**Affiliations:** a School of nursing, Lanzhou University; b Department of nursing, Gansu Provincial Hospital, China.

**Keywords:** teaching method, the critical thinking ability, autonomous learning ability

## Abstract

**Background:**

One of the major challenges in nursing and medical education is to foster the critical thinking ability and autonomous learning ability for students. But the effect of different teaching methods on these abilities of nursing or medical students has not been conclusive, and few studies have directly compared the differences in the effects of different teaching methods. As a result, it is necessary for students to evaluate the impact of different teaching methods on critical thinking ability and autonomous learning ability.

**Methods:**

A systematic search will be performed using Chinese National Knowledge Infrastructure, Wanfang Data (Chinese database), VIP Information (Chinese database), Chinese Biomedical Literature, and English language databases, including PubMed and Embase, Web of Science, CINAHL Complete (EBSCO0, Cochrane library to identify relevant studies from inception to July 10, 2020. We will include random controlled trials that evaluated the different teaching methods. The Quality Assessment of Diagnostic Accuracy Studies 2 quality assessment tool will be used to assess the risk of bias in each study. Standard pairwise meta-analysis and network meta-analysis will be performed using STATA V.12.0, MetaDiSc 1.40, and R 3.4.1 software to compare the diagnostic efficacy of different hormonal biomarkers.

**Results:**

The results of this study will be published in a peer-reviewed journal.

**Conclusion:**

This study will summarize the direct and indirect evidence to determine the effectiveness of different teaching methods for medical or nursing students and attempt to find the most effective teaching method.

**Ethics and dissemination:**

Ethics approval and patient consent are not required, because this study is a meta-analysis based on published studies.

**INPLASY registration number:**

INPLASY202070017

## 1 Introduction

The ability of critical thinking is to be deliberate about thinking and actively assess and regulate one's cognition,[^1^,^2^] which is vital for nursing students and medical students which prepare them for clinical practice,^[3]^ because critical thinking makes them respond quickly to patients’ urgent problems, make a best clinical decision, and provide safe and quality care.^[4]^ And then, students with clinical thinking skills have capabilities such as information seeking, data analysis, decision making, and feedback.^[5]^ However, Absent critical thinking, 1 typically relies on heuristics, a quick method or shortcut for problem-solving, and can fall victim to cognitive biases.^[6]^ Cognitive biases can lead to diagnostic errors, which result in increased patient morbidity and mortality, and the adverse event of nursing.^[7]^ Therefore, critical thinking and experience with technology have been noted as important qualities for graduates transitioning into professional roles^[8]^

The representative of Social Cognitive School, American psychologist Bandura^[9]^ believes that autonomous learning is that learners constantly monitor and adjust their cognitive and emotional states, observe and apply various learning strategies, adjust learning behaviors, and strive to create and use the process of using material and social resources that contribute to learning. Autonomous learning is defined as a process where the learner is motivationally, behaviourally and meta-cognitively proactive in the learning process.^[10]^ Besides, in the clinical environment, autonomous learning has been linked with academic achievement,[^11^,^12^,^13^] success in clinical skills,^[14]^ and emotional health.^[15]^ However, 1 of the major challenges in nursing and medical education is to develop an effective teaching method to foster critical thinking and autonomous learning ability for students.

In medical education, different teaching methods have different effects on nursing or medical students’ critical thinking and autonomous learning ability. In addition, more and more medical educators have recognized the shortcomings of traditional teaching methods, so they try to use a variety of teaching methods to enhance students’ critical thinking and autonomous learning ability, for example, case-based learning, problem-based learning, simulation-based learning. Compared with traditional teaching methods, these teaching methods reflect their own advantages. At present, the effect of different teaching methods on the critical thinking and autonomous learning ability of nursing or medical students has not been conclusive, and few studies have directly compared the differences in the effects of different teaching methods. Consequently, it is necessary and practical to evaluate the impact of different teaching methods on the critical thinking ability and autonomous learning ability of nursing or medical students

## 2 Methods

A network meta-analysis (NMA) will be conducted to test the differences of different teaching methods. We have registered the protocol on the International Platform of Registered Systematic Review and Meta-analysis Protocols (INPLASY),^[16]^ and the registration number was INPLASY202070017. We will follow the Preferred Reporting Items for Systematic Reviews and Meta-Analyses statements^[17]^ to report our NMA.

### 2.1 Eligibility criteria

#### 2.1.1 Types of patients

Medical students or nursing students will be included. There will also be no restrictions based on other conditions, such as age, educational attainment, gender, different courses.

#### 2.1.2 Types of studies

We will consider only randomized controlled trials (RCTs) of teaching methods for medical or nursing students. We will exclude non-RCTs, quasi-RCTs, uncontrolled trials, and reviews.

#### 2.1.3 Types of interventions

Studies that evaluated any kind of teaching method (case-based learning, problem-based learning, simulation-based learning) will be included. We will exclude trials that teaching methods are not used as a major therapy. The control interventions will include traditional teaching.

#### 2.1.4 Types of outcome measures

##### 2.1.4.1 Primary outcomes

The primary outcomes are critical thinking (CT), autonomous learning ability. And CT was evaluated by the California Critical Thinking Disposition Inventory (CCTDI),^[18]^ and autonomous learning ability was evaluated by the Self-Directed Learning Instrument (SDLI) for Nursing Students.^[19]^

##### 2.1.4.2 Secondary outcomes

The secondary outcome measures will include:

1. student satisfaction: Undergraduate Nursing Student Academic Satisfaction Scale^[20]^
2. score: Self-made scale based on different research content

### 2.2 Search methods and the identification of studies

#### 2.2.1 Electronic searches

**Figure 1:**
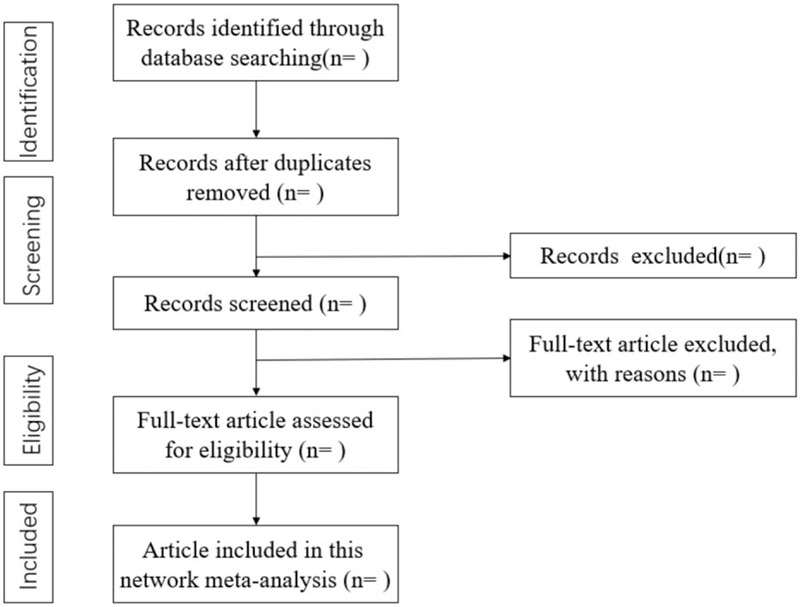
Flow chart of searching and screening studies.

We searched Chinese National Knowledge Infrastructure, Wanfang Data (Chinese database), VIP Information (Chinese database), Chinese Biomedical Literature, and English language databases, including PubMed and Embase, Web of Science, CINAHL Complete (EBSCO), Cochrane library to July 10, 2020, for different teaching methods. The search term will include 3 parts: that is teaching methods (Training Technique∗; Training Technic∗; Problem Based Learning; Problem-Based Curriculum; Problem Based Curriculum; Problem-Based Curricula; Problem Based Curricula; Experiential Learning; Active Learning; Self Directed Learning as Topic; Simulation Training; Interactive Learning; Interactive Learning), critical thinking or autonomous learning ability (Thinking Skill∗, Thought∗, Critical Thinking, Independent learning capability, autonomous learning ability, Self-learning ability, Learner Autonomy, Self-regulated ability), and medical students or nursing students (Medical Student∗ OR Pupil Nurse∗ OR Nursing Student^∗^). The equivalent search entries will be used while searching in Chinese databases. The fully reproducible search strategy provided in Table 1 is for PubMed. This will be appropriately adapted for search in the other databases. And the flow chart of searching and screening studies is showed at Fig. 1

**Table 1:**
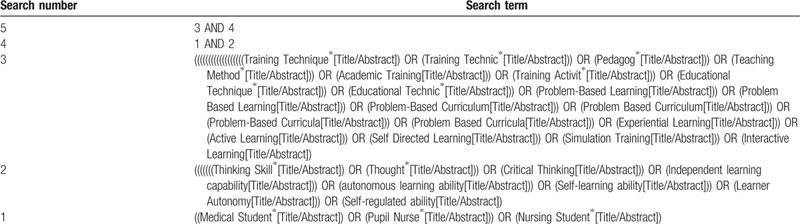
Search strategy used in the PubMed database.

#### 2.2.2 Searching other resources

In addition, we will also search for dissertations and grey literature to identify systematic reviewes or clinical trials related to teaching methods. Besides, related journals and conference processes will be manually searched.

### 2.3 Data collection and analysis

#### 2.3.1 Selection of studies and data extraction

Initial search records will be imported into ENDNOTE X9 literature management software, then the titles and abstracts of records will be screened to identify potential trials according to eligibility criteria. Next, full-text versions of all potentially relevant trials will be obtained and reviewed to ensure eligibility.

A standard data extraction form will be created using Microsoft Excel 2013 to collect data of interest. Which include eligible studies characteristics (eg, name of the first author, year of publication, the country in which the study was conducted), intervention characteristics (eg, the name of teaching methods, intervention time, time of duration), population characteristics (eg, gender, mean age, sample) and outcome(eg, CT, autonomous learning ability, student satisfaction, score)

Study selection and data extraction will be performed by 1 reviewer (YB), and will be checked by other reviewers (CYT, SQ). Any conflicts will be resolved by discussion.

#### 2.3.2 Assessment of risk of bias

Two reviewers (Y.B. and C.YT.) will independently assess the risk of bias for each study as low, moderate, or high using the Quality Assessment of Diagnostic Accuracy Studies.^[21]^ And conflicts will be also resolved by discussion.

#### 2.3.3 Geometry of the network

Using R software V.3.4.1, a network plot will be drawn. In network plots, the size of the nodes is proportional to the number of studies evaluating a test, and the thickness of the lines between the nodes is proportional to the number of direct comparisons between tests. The network is connected because there exists at least 1 study evaluating a given test together with at least 1 of the other remaining tests.^[22]^ A loop connecting 3 tests indicates that there is at least 1 study comparing the 3 targeted tests simultaneously.

#### 2.3.4 NMA

##### 2.3.4.1 Pairwise meta-analyses

We will construct forest plots showing estimates of sensitivity, specificity, positive likelihood ratio, negative likelihood ratio, diagnostic odds ratio, and their corresponding 95% confidence intervals for each index test using STATA V.12.0 (Stata) and MetaDiSc 1.40. *Q* value and the inconsistency index (I2 test) will estimate the heterogeneity of each study. If the *I* ^2^ is ≤50%, it means that statistical heterogeneity could be negligible, and the fixed effects model will be used. If the *I* ^2^ is >50%, we will explore sources of heterogeneity by subgroup analysis and meta-regression. If there is no clinical heterogeneity, the random-effects model will be used to perform the meta-analysis. In addition, we will also plot sensitivities and specificities in the summary receiver operating characteristic space, using different symbols for different hormonal biomarkers. Besides, we will use STATA V.12.0 (Stata) and Review Manager 5.30 (RevMan) analysis software to build the hierarchical summary receiver operating characteristic graphics for each index test.

##### 2.3.4.2 Indirect comparisons between competing diagnostic tests

We could use STATA V.12.0 (Stata) software to calculate relative diagnostic outcomes between index tests, including relative sensitivity, relative specificity, relative diagnostic odds ratio, relative positive likelihood ratio, and relative negative likelihood ratio, and then we could use these outcomes to conduct indirect comparisons.

##### 2.3.4.3 Subgroup analysis

If sufficient studies are available, we will explore meta-regression or subgroup analysis based on the age, intervention time, and duration of intervention; the country in which the study was conducted, and the risk factors of teaching methods.

## 3 Discussion

To the best of our knowledge, this is the first NMA protocol comparing different teaching methods for nursing or medical students to foster the critical thinking ability and autonomous learning ability with RCTs. The study will provide a ranking of mesh fixation for teaching methods and we hope the result will provide recommendations for education managers to foster students’ ability. This protocol is designed in adherence to guidelines for NMA protocols and will be conducted and reported strictly according to the PRISMA extension statement for NMA.^[23]^

## Acknowledgments

We are grateful for the helpful reviewer comments on this paper.

## Author contributions

Bei Yun, Yi-Tong Cai, and QianSu: plan and design the research. BeiYun, Yi-Tong Cai, Qian Su, and Lin Han tested the feasibility of the study. Yi-Tong Cai, Qian Su, Lian Chen, Chao-Ran Qu and Lin Han provided methodological advice, polished, and revised the manuscript. Bei Yun and Qian Su wrote the manuscript; all authors approved the final version of the manuscript.

**Competing interests** None declared

**Conceptualization:** Lin Han.

**Investigation:** Lin Han.

**Methodology:** Bei Yun, YiTong Cai, QianSu, LianChen, Chaoran Qu and LinHan

**Provenance and peer review**

**Resources:** Bei Yun, YiTong Cai, QianSu.

**Software:** Bei Yun, YiTong Cai.

**Supervision:** QianSu, LinHan.

**Validation:** Lian Chen and ChaoRan Qu

**Writing – original draft:** Bei Yun,Qian Su.

**Writing – review & editing:** Bei Yun, Qian Su.

Not commissioned; externally peer reviewed.

## References

[1] Huang GC Newman LR Schwartzstein RM Critical thinking in health professions education: summary and consensus statements of the Millennium Conference 2011. Teach Learn Med 2014;26:95–102. 24405353 10.1080/10401334.2013.857335

[2] Croskerry P From mindless to mindful practice--cognitive bias and clinical decision making. N Engl J Med 2013;368:2445–8. 23802513 10.1056/NEJMp1303712

[3] Kaya H Şenyuva E Bodur G Developing critical thinking disposition and emotional intelligence of nursing students: a longitudinal research. Nurse Educ Today 2017;48:72–7. 27721088 10.1016/j.nedt.2016.09.011

[4] Kaddoura MA Van Dyke O Yang Q Correlation between critical thinking skills and national council licensure examination for registered nurses success in accelerated bachelor nursing students. Teach Learn Nurs 2017;12:3–7.

[5] Von Colln-Appling C Giuliano D A concept analysis of critical thinking: a guide for nurse educators. Nurse Educ Today 2017;49:106–9. 27902948 10.1016/j.nedt.2016.11.007

[6] Tversky A Kahneman D Judgment under uncertainty: heuristics and biases. Science (New York, NY) 1974;185:1124–31. 10.1126/science.185.4157.1124 17835457

[7] Croskerry P The importance of cognitive errors in diagnosis and strategies to minimize them. Acad Med 2003;78:775–80. 12915363 10.1097/00001888-200308000-00003

[8] Borglin G Fagerström C Nursing students’ understanding of critical thinking and appraisal and academic writing: a descriptive, qualitative study. Nurse Educ Pract 2012;12:356–60. 22633117 10.1016/j.nepr.2012.04.009

[9] Zimmerman BJ Risemberg R Self-regulatory dimensions ofacademic learning and motivationM]//Phye G D. Hand-book of academic learning. 1997;San Diego: Academic Press, 105-125.

[10] Zimmerman BJ Becoming a self-regulated learner: which are the key subprocesses? Contemp Educ Psychol 1986;11:307–13.

[11] Artino ARJ Dong T DeZee KJ Achievement goal structures and self-regulated learning: relationships and changes in medical school 2012;87:1375–81. 10.1097/ACM.0b013e3182676b55 22914521

[12] Turan S Konan A Self-regulated learning strategies used in surgical clerkship and the relationship with clinical achievement. J Surg Educ 2012;69:218–25. 22365869 10.1016/j.jsurg.2011.09.003

[13] Song HS Kalet AL Plass JL Assessing medical students’ self-regulation as aptitude in computer-based learning. Adv Health Sci Educ Theory Pract 2011;16:97–107. 20872071 10.1007/s10459-010-9248-1 PMC3828293

[14] Cleary TJ Sandars J Assessing self-regulatory processes during clinical skill performance: a pilot study. Med Teach 2011;33:e368–74. 21696270 10.3109/0142159X.2011.577464

[15] Van Nguyen H Laohasiriwong W Saengsuwan J The relationships between the use of self-regulated learning strategies and depression among medical students: an accelerated prospective cohort study. Psychol Health Med 2015;20:59–70. 24628063 10.1080/13548506.2014.894640

[16] Yun et al. International Platform of Registered Systematic Review and Meta-analysis Protocols. Inplasy protocol 202070017. doi:10.37766/inplasy2020.7.0017. Available at: https://inplasy.com/.

[17] Moher D Liberati A Tetzlaff J Preferred reporting items for systematic reviews and meta-analyses: the PRISMA statement. Int J Surg (London, England) 2010;8:336–41. 10.1016/j.ijsu.2010.02.007 20171303

[18] Facione NC Facione PA Sanchez CA Critical thinking disposition as a measure of competent clinical judgment: the development of the California Critical Thinking Disposition Inventory. J Nurs Educ 1994;33:345–50. 7799093 10.3928/0148-4834-19941001-05

[19] Cheng SF Kuo CL Lin KC Development and preliminary testing of a self-rating instrument to measure self-directed learning ability of nursing students. Int J Nurs Stud 2010;47:1152–8. 20223455 10.1016/j.ijnurstu.2010.02.002

[20] Dennison S El-Masri MM Development and psychometric assessment of the undergraduate nursing student academic satisfaction scale (UNSASS). J Nurs Meas 2012;20:75–89. 22988780 10.1891/1061-3749.20.2.75

[21] Whiting PF Rutjes AW Westwood ME QUADAS-2: a revised tool for the quality assessment of diagnostic accuracy studies. Ann Intern Med 2011;155:529–36. 22007046 10.7326/0003-4819-155-8-201110180-00009

[22] Nyaga VN Aerts M Arbyn M ANOVA model for network meta-analysis of diagnostic test accuracy data. Stat Methods Med Res 2018;27:1766–84. 27655805 10.1177/0962280216669182

[23] Hutton B Salanti G Caldwell DM The PRISMA extension statement for reporting of systematic reviews incorporating network meta-analyses of health care interventions: checklist and explanations. Ann Intern Med 2015;162:777–84. 26030634 10.7326/M14-2385

